# Comparison of Recombination Rate, Reference Bias, and Unique Pangenomic Haplotypes in *Cannabis sativa* Using Seven De Novo Genome Assemblies

**DOI:** 10.3390/ijms26031165

**Published:** 2025-01-29

**Authors:** George M. Stack, Michael A. Quade, Dustin G. Wilkerson, Luis A. Monserrate, Philip C. Bentz, Sarah B. Carey, Jane Grimwood, Jacob A. Toth, Seth Crawford, Alex Harkess, Lawrence B. Smart

**Affiliations:** 1 Horticulture Section, School of Integrative Plant Science, Cornell University, Geneva, NY 14456, USA; gms252@cornell.edu (G.M.S.); maq28@cornell.edu (M.A.Q.); dgw65@cornell.edu (D.G.W.); lam382@cornell.edu (L.A.M.); jat363@cornell.edu (J.A.T.); 2 HudsonAlpha Institute for Biotechnology, Huntsville, AL 35806, USA; pbentz@hudsonalpha.org (P.C.B.); scarey@hudsonalpha.org (S.B.C.); jgrimwood@hudsonalpha.org (J.G.); aharkess@hudsonalpha.org (A.H.); 3 Oregon CBD, Independence, OR 97351, USA; seth@jackhempicine.com

**Keywords:** hemp, *Cannabis*, genome assembly, synteny, *k*-mer, recombination, reference bias

## Abstract

Genomic characterization of *Cannabis sativa* has accelerated rapidly in the last decade as sequencing costs have decreased and public and private interest in the species has increased. Here, we present seven new chromosome-level haplotype-phased genomes of *C. sativa*. All of these genotypes were alive at the time of publication, and several have numerous years of associated phenotype data. We performed a *k*-mer-based pangenome analysis to contextualize these assemblies within over 200 existing assemblies. This allowed us to identify unique haplotypes and genomic diversity among *Cannabis sativa* genotypes. We leveraged linkage maps constructed from F_2_ progeny of two of the assembled genotypes to characterize the recombination rate across the genome showing strong periphery-biased recombination. Lastly, we re-aligned a bulk segregant analysis dataset for the major-effect flowering locus *Early1* to several of the new assemblies to evaluate the impact of reference bias on the mapping results and narrow the locus to a smaller region of the chromosome. These new assemblies, combined with the continued propagation of the genotypes, will contribute to the growing body of genomic resources for *C. sativa* to accelerate future research efforts.

## 1. Introduction

*Cannabis sativa* L. has long been cultivated by humans for its durable fiber, nutritious seeds, and culturally and medicinally valuable secondary metabolites. Thought to have originated in Central or East Asia, *C. sativa* now has a global distribution [1,2]. As the range of *C. sativa* expanded, natural and human selection shaped genotypic and phenotypic differentiation, adapting populations for use in different market classes and local environments. Research on *C. sativa* has been restricted as a result of the plant’s decades-long classification as a controlled substance but has accelerated in recent years as regulations have been lifted on the cultivation of hemp and marijuana in many jurisdictions. Among those areas of research, *C. sativa* genetics and genomics have rapidly expanded. In the decade after the first *C. sativa* genome assembly was published by van Bakel et al. [3], numerous assemblies have been published [4,5,6]. These resources enabled significant progress in various areas of genetic and genomic research, including quantitative trait locus (QTL) mapping [7,8,9,10,11,12], genome-wide association studies (GWAS) [13,14,15,16], phylogenetics and population structure [2,17,18,19], and gene family characterization [20,21,22]. Two emerging challenges for the *C. sativa* research community are to (1) comprehensively capture the genomic diversity of the species and (2) reconcile and leverage the large amounts of genomic data to make meaningful contributions to fundamental research and commercial production systems.

It is well established that genome assemblies of one or a few individuals are insufficient to capture the genetic diversity of a species [23,24]. There has been a dramatic rise of high-quality publicly available *C. sativa* genome assemblies in the past year, from less than 20 to more than 200, including more than 40 chromosome-level phased diploid assemblies [25,26]. As we move into the era of *C. sativa* pangenomics, we will be able to simultaneously consider diverse haplotypes from all of these assemblies when analyzing genetic and genomic data.

As the *C. sativa* genomics research community works towards a comprehensive pangenome, sequencing and assembly of Y chromosomes will be essential for accurately characterizing sex determination systems and exploring their evolutionary dynamics. *Cannabis sativa* is predominately dioecious, with a long-established XY sex determination system, where males are heterogametic [27]. It shares this XY system with its sister taxon, hop (*Humulus* spp.), both members of the Cannabaceae [1]. The first assembled *C. sativa* Y chromosomes were made publicly available in 2024 [25,26]. Despite this progress, there are still less than 10 assembled Y chromosome sequences compared to the dozens of assembled X chromosome sequences.

In addition to sampling sex chromosome diversity, targeted sampling will be necessary to capture the global diversity of *C. sativa* germplasm. There is evidence of strong population structure in *C. sativa*, both associated with geographic origin and post-harvest market class (Table 1). Over the past decade, studies have broadly differentiated between high-cannabinoid germplasm and European fiber/grain hemp germplasm, which some taxonomists classify as two subspecies, subsp. *indica* and subsp. *sativa*, respectively [28,29]. The terms “marijuana” or “drug-type” are commonly used to encompass all high-cannabinoid *C. sativa*, where “hemp” is used to group primarily European grain and fiber germplasm. In addition to separating these two groups broadly, some studies resolve additional population structure within them (Table 1). Distinct sub-populations of Asian hemp germplasm, more closely related to high-cannabinoid germplasm than European hemp germplasm, as well as United States (U. S.) feral hemp, which is sometimes grouped with European fiber hemp, have been repeatedly identified [18,19]. Several studies have proposed additional groups of wild and feral material from Asia, but this region is severely under-sampled, especially as the presumed center of origin and domestication [1,2]. Considering this genetic diversity, the majority of the assembled genome sequences are from a relatively narrow group of high-cannabinoid genotypes, many of which are breeding lines sampled from the Oregon CBD (OCBD) program [25]. Assemblies of related individuals will be valuable for phasing and tracking the inheritance of different haplotypes, but additional sampling of diverse germplasm will be necessary to fully capture the genetic diversity of the species.

One result of capturing diverse haplotypes in high-quality assemblies will be a more robust analysis of new genetic and genomic data. A common challenge in these analyses is the significant reference bias that arises when mapping reads to different genome assemblies. This bias occurs in part because reads derived from reference haplotypes achieve higher quality mapping than those derived from non-reference haplotypes [32,33]. Strategies to mitigate this bias include the development of pangenome graphs as references [24,34] and comparison of alignments using multiple high-quality haploid assemblies as references [35]. The availability of diverse genome assemblies will also provide resources for the selection of targets for molecular markers in genome editing.

Assemblies that capture diverse haplotypes can also accelerate plant breeding by positioning beneficial and deleterious haplotypes and providing information about linkage disequilibrium (LD) among haplotypes. Plant breeders rely on meiotic recombination to break LD and generate novel haplotypes that can be selected on to develop improved cultivars [36]. There is growing interest in the potential of modifying the frequency and location of crossover events to improve selection efficiency [36,37]; however, plant breeders are largely limited by the existing recombination landscape. Although limited data have been published investigating variation in recombination rates across the *C. sativa* genome, analyses with assemblies of ‘Purple Kush’ and ‘FINOLA’ found bias towards the gene-rich, repeat-poor regions at chromosome ends [4].

The objectives of this study were to: (1) construct high-quality genome assemblies for seven new *Cannabis sativa* genotypes, including two XY staminate individuals; (2) analyze these new genome assemblies in the context of existing assemblies to identify unique haplotypes; (3) utilize two of these assemblies, representing the parents of a bi-parental F_2_ mapping population, to characterize the recombination landscape of *C. sativa*; and (4) leverage the new genome assemblies to investigate the impact of reference bias on bulk segregant analysis (BSA) for trait mapping.

## 2. Results

### 2.1. Genome Assemblies Are High-Quality and Contiguous

For the seven genotypes sequenced and assembled (Table 2), the average assembly size was 754 Mb for haplotypes with chromosome X (ChrX) and 782 Mb for haplotypes with chromosome Y (ChrY) (Supplementary Table S1). The haplotypes with ChrY were 6.02% and 3.93% larger than the haplotypes with ChrX for the GVA-H-21-1003-002 and GVA-H-22-1061-002 assemblies, respectively. The number of scaffolds not incorporated into chromosomes ranged from 58 to 469, comprising between 1.40% and 3.55% of the assemblies (Supplementary Table S1). The number of structural annotations identified by Helixer [38] ranged from 23,182 to 28,296 with a mean of 24,752 (Supplementary Table S1). BUSCO scores for raw assembly FASTAs ranged from 96 to 98.5, and for Helixer-predicted proteins from 95.7 to 98.1 (Supplementary Table S1). BUSCO scores were consistently lower for haplotypes with ChrY compared to their counterparts with ChrX. In the GVA-H-22-1061-002 and GVA-H-21-1003-002 assemblies, 49 BUSCOs were identified in each of the ChrX-specific regions, but only 5 and 4 were identified in the ChrY sex-determining regions (SDRs), respectively.

### 2.2. Phased Chromosome-Level Assemblies Cluster in Agreement with Established Population Structure

**Figure 1:**
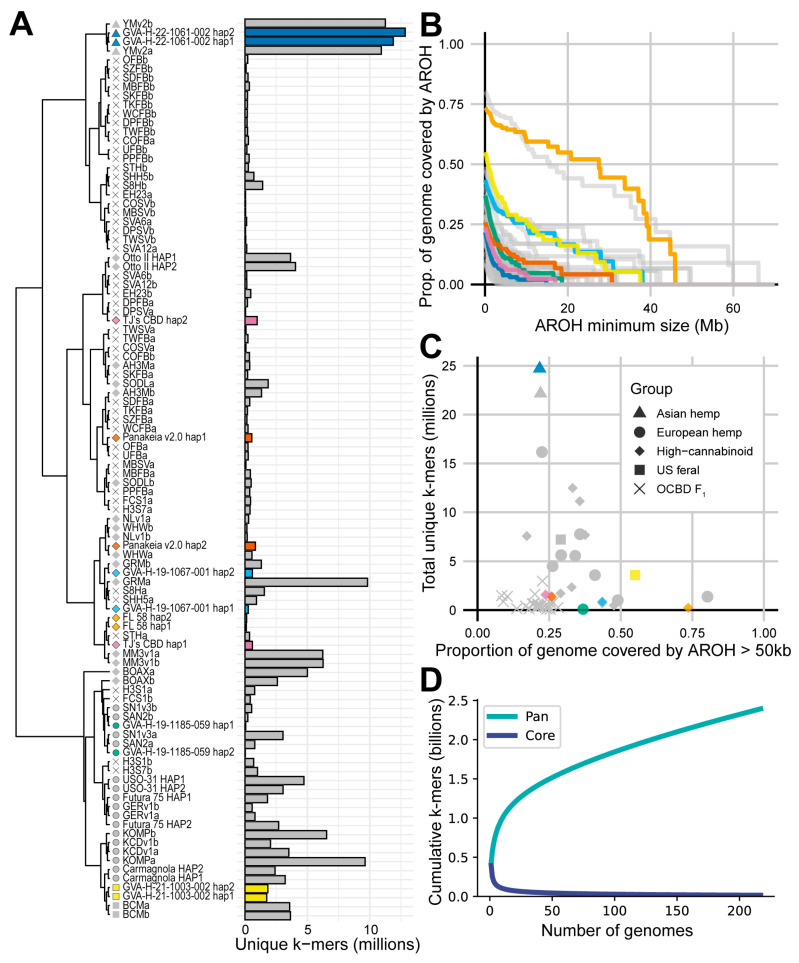
Seven new *C. sativa* assemblies in the context of existing genomes (**A**) Jaccard clustering of 100 haplotypes from 50 chromosome-level phased assemblies based on a 31-mer PanKmer index. An aligned bar plot shows the number of unique 31-mers identified in each haplotype from a 31-mer index of 217 *C. sativa* assemblies. (**B**) The proportion of each of the 50 chromosome-level phased *C. sativa* assemblies covered by aligned runs of homozygosity (AROH) of various sizes derived from pairwise minimap2 alignments between haplotypes (**C**) The number of unique *k*-mers found in both haplotypes plotted against the proportion of the genome covered by AROH greater than 50 kb. (**D**) PanKmer collector’s curve from the 31-mer index of 217 *C. sativa* assemblies. Colors in panels (**A**–**C**) indicate assemblies from this paper. Shapes in panels (**A**,**C**) indicate the name and group that each assembly is associated with based on provided metadata per the legend in panel (**C**).

Jaccard clustering based on a PanKmer [39] index of 50 phased chromosome-level assemblies broadly separated the high-cannabinoid assemblies from the European hemp assemblies (Figure 1A, Supplementary Figure S1) in accordance with previous population structure analyses conducted in *C. sativa* (Table 1). Within the high-cannabinoid group, the Asian hemp assemblies, ‘YunMa’ (YMv2a, YMv2b) and GVA-H-22-1061-002, formed a distinct cluster adjacent to a set of OCBD F_1_ assemblies. Likewise, the U.S. feral assemblies, Boone County (BCMa, BCMb) and GVA-H-21-1003-002, formed a subgroup within the European hemp cluster, positioned near the Italian fiber cultivar ‘Carmagnola’. Both haplotypes of the high-CBD cultivar ‘BoAx’ (BOAXa, BOAXb) loosely clustered with the European hemp assemblies. Apart from ‘Panakeia v2.0’ hap1 and ‘TJ’s CBD’ hap 2, most haplotypes of the new high-cannabinoid assemblies presented in this study clustered near each other.

**Figure 2:**
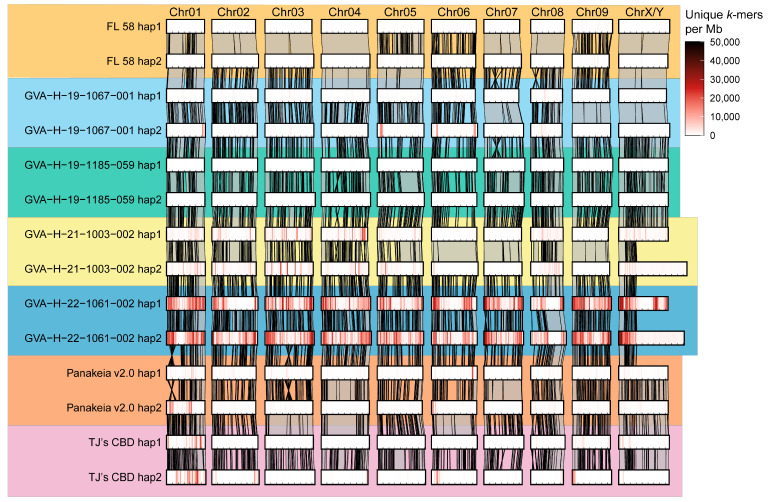
Alignment of seven phased chromosome-level genome assemblies of *C. sativa*. Each sequenced genotype is represented by 20 chromosomes phased into two haploid assemblies. Shading within each rectangle indicates the count of unique 31-mers aligned in 1 Mb bins across each assembled chromosome. Gray polygons with black borders connecting homologous chromosomes show pairwise alignments greater than 400 kb. For the two XY assemblies, GVA-H-21-1003-002 and GVA-H-22-1061-002, the Y chromosome is included in haplotype 2.

Unique *k*-mer density also varies among chromosomes and assemblies, with greater densities indicating haplotypes that are unique among the assemblies included in the PanKmer index. Some assemblies, like GVA-H-19-1067-001 hap2 and ‘Panakeia v2.0’ hap1, contain isolated chromosomal regions with greater densities of unique *k*-mers (Figure 2). In contrast, both haplotypes of the GVA-H-22-1061-002 assembly have large numbers of unique *k*-mers across the majority of every chromosome (Figure 2). Within both haplotypes containing ChrY, the pseudoautosomal region (PAR) has a greater density of unique *k*-mers than the SDR (Figure 2). 

**Table 2:** Description of sequenced genotypes. Phenotypic sex is classified as either pistillate (P), staminate (S), or monoecious (Mo). Sex chromosomes are classified as either XX or XY. Chemotype is classified as CBD-dominant (III) or CBG-dominant (IV) as defined by de Mandolino and Carboni [40]. Entity or institution from which genotype was provided. If available, the National Plant Germplasm System (NPGS) accession is provided. The amounts of raw sequence data generated by each platform are provided in gigabases (Gb) and number of reads. References are provided for studies where the genotypes were used. * paired 2 × 150 bp reads.

Genotype	Sex	Chemotype	Source	U.S. NPGS Accession	PacBio HiFi Data (Gb/Reads)	Omni-C^®^ Data (Gb/Reads *)	Refs.
‘FL 58’	P/XX	III	Sunrise Genetics	G 33236	17.9/1.25 M	126.8/43.0 M	[10,18,41,42,43,44]
‘Panakeia v2.0’	P/XX	IV	Bazelet	-	16.9/1.09 M	107.8/37.0 M	[10]
‘TJ’s CBD’	P/XX	III	Stem Holdings Agri	G 33580	29.5/2.62 M	128.2/43.7 M	[10,18,41,42,43,44,45,46,47,48]
GVA-H-19-1067-001	P/XX	III	Cornell Hemp Breeding Program	-	19.3/1.18 M	111.8/38.3 M	[10,44]
GVA-H-19-1185-059	Mo/XX	IV	Cornell Hemp Breeding Program	-	33.9/2.17 M	86.2/29.3 M	[10]
GVA-H-21-1003-002	S/XY	III	Cornell Hemp Breeding Program	Derived from G 33199	21.9/2.66 M	92.3/31.6 M	[10]
GVA-H-22-1061-002	S/XY	III	Cornell Hemp Breeding Program	Derived from G 33545	24.3/1.50 M	81.9/28.0 M	[10]

### 2.3. Assemblies Vary in Number and Position of Unique k-mers

Unique *k*-mers were extracted from a PanKmer index of 217 *C. sativa* assemblies. The PanKmer collector’s curve (Figure 1D) shows a continued increase in the cumulative number of *k*-mers as more assemblies are added to the index. The number of unique *k*-mers in each assembly was somewhat associated with the population or diversity group origin of the assembly. Overall, high-cannabinoid assemblies, particularly the OCBD F_1_ assemblies, had lower numbers of unique *k*-mers, most with fewer than 1 million per haplotype (Figure 1A, Supplementary Table S2). In contrast, the European and U.S. feral assemblies tended to have a moderate number of unique *k*-mers, with most between 1.5 and 8 million per haplotype (Figure 1A, Supplementary Table S2). The Asian hemp assemblies had the greatest counts, with greater than 10 million unique *k*-mers in each haplotype (Figure 1A, Supplementary Table S2). The two additional phased contig-level Asian hemp assemblies, YMMv1 and CNBv1, further illustrate this with particularly large counts of 28.6 million and 43.2 million unique *k*-mers, respectively (Supplementary Table S2).

### 2.4. Pairwise Haplotype Alignment Can Be Used as a Metric for Homozygosity

To investigate the position and coverage of putatively homozygous stretches in phased chromosome-level assemblies, minimap2 [49] alignments were filtered to identify aligned runs of homozygosity (AROH). The assemblies show substantial variation in the proportion of the genome covered by progressively larger AROH (Figure 1B); some assemblies lack AROH larger than 20 Mb, while others have up to 50% of their genome covered by AROH of 30 Mb or larger. Notably, two assemblies, ‘FL 58’ and GERv1, exhibit extensive AROH coverage, suggesting a high level of inbreeding. As visualized in Figure 2, ‘FL 58’ shows nearly complete AROH coverage on Chr02, 03, 04, 07, 08, and X. Similarly, chromosome-spanning AROH are found in GVA-H-21-1003-002 on Chr06, 07, and 09, as well as GVA-H-19-1067-001 on Chr07, 09, and X (Figure 2).

Additional insights into highly heterozygous assemblies emerge when the number of unique *k*-mers per assembly is plotted against AROH coverage greater than 50 kb (Figure 1C). Asian hemp GVA-H-22-1061-002 and ‘YunMa’ (YMv2a, YMv2b), along with the Hungarian cultivar ‘Kompolti’ (KOMPa, KOMPb), have high heterozygosity, as evidenced by low AROH coverage, and have large numbers of unique *k*-mers. In contrast, the OCBD F_1_ assemblies, despite showing similar or greater levels of heterozygosity, contain dramatically fewer unique *k*-mers (Figure 1C).

### 2.5. Chromosomes Show Strong Periphery Bias in Recombination Rate

**Figure 3:**
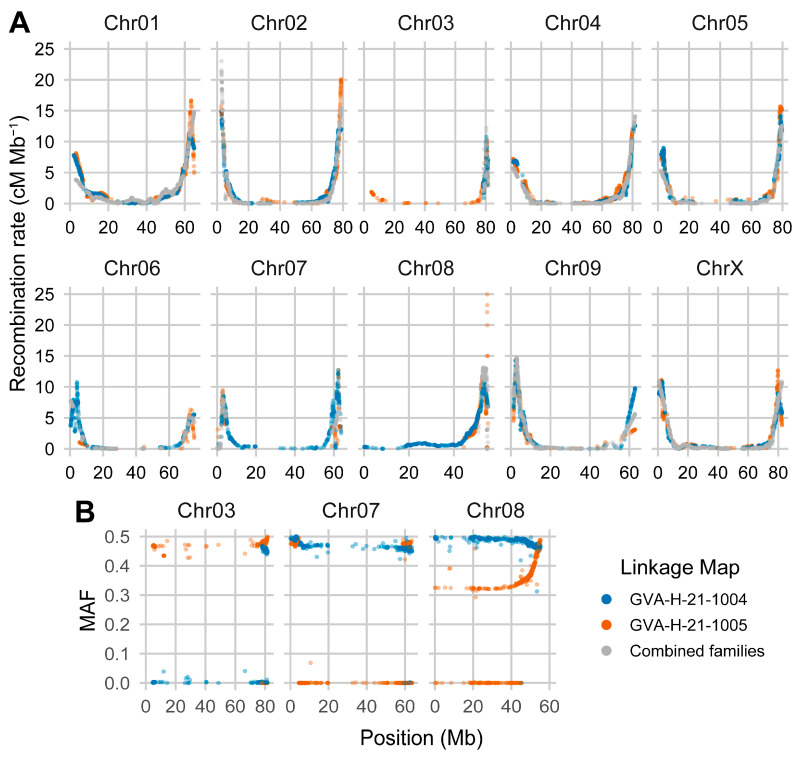
Estimated recombination rate across the 10 chromosomes of *C. sativa*. (**A**) Recombination rate plotted against position. Colors in both panels represent the linkage maps for each of the ‘FL 58’ × ‘TJ’s CBD’ F_2_ families as well as the map from the combined data of both families. (**B**) Minor allele frequency (MAF) plotted against position on Chr03, Chr07, and Chr08 for the two F_2_ families.

Marker sequences from the GVA-H-21-1004, GVA-H-21-1005, and combined-family linkage maps were aligned to assemblies of the parental genotypes ‘FL 58’ and ‘TJ’s CBD’ [10] to estimate recombination rates across the genome. Analyses with all three linkage maps showed agreement in estimated recombination rates across the genome (Figure 3A). Recombination is significantly suppressed in the center of all 10 chromosomes (Figure 3A), likely due to structural constraints and chromatin compaction, with most crossovers occurring near telomeres. Most chromosomes have regions 40 Mb or larger with recombination rates less than 0.5 cM per Mb (Figure 3A). In the GVA-H-21-1004 family, map recombination appears suppressed across the entire short arm of Chr08 (Figure 3A).

### 2.6. Segregation Distortion and Runs of Monomorphic Markers Restrict Linkage Map Coverage

Aligning marker-associated sequences from the ‘FL 58’ × ‘TJ’s CBD’ F_2_ linkage maps to parental genomes provides a detailed view of how well the linkage groups cover the physical map. Three chromosomes—Chr03, Chr07, and Chr08—have large gaps in coverage in either the GVA-H-21-1004 or GVA-H-21-1005 F_2_ family maps, as well as the combined map using both families (Figure 3A). Plotting the minor allele frequency (MAF) of markers across these chromosomes highlights two major factors that limited linkage group construction (Figure 3B). Specifically, Chr03 in the GVA-H-21-1004 family and Chr07 in the GVA-H-21-1005 family contain long stretches of monomorphic markers, while the majority of Chr08 in the GVA-H-21-1005 family displays significant segregation distortion.

### 2.7. Bulk Segregant Analysis Mapping of Early1

**Figure 4:**
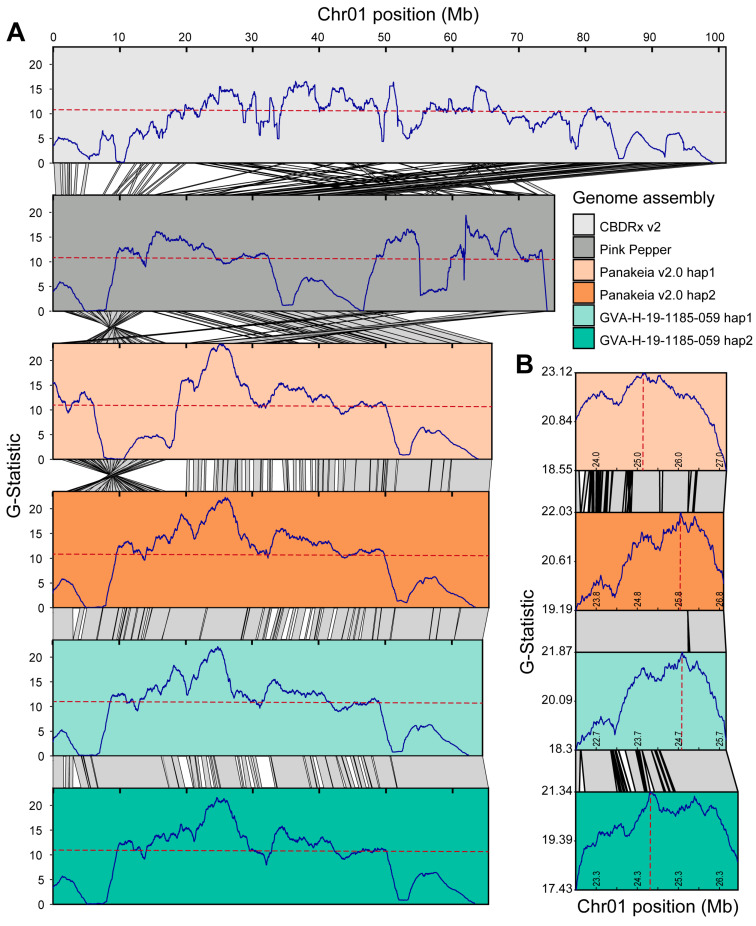
Bulk segregant analysis (BSA) mapping of the major-effect flowering locus *Early1* using six different genome assemblies as references for alignment. (**A**) Moving average of G-statistic, dark blue line, in sliding windows every 10 kb plotted against position for Chr01. Dashed red horizontal lines are the significance threshold. Gray polygons with black borders connecting chromosomes show pairwise minimap2 alignments > 250 kb. (**B**) Syntenic ~3.5 Mb section of Chr01 surrounding the peak markers when using the ‘Panakeia v2.0’ and GVA-19-1185-059 assemblies. Dashed red vertical lines are the most significant sliding window when using each assembly. Gray polygons with black borders connecting homologous chromosomes show pairwise minimap2 alignments > 5 kb.

Alignment of Chr01 from CBDRx v2 (cs10, NCBI GenBank GCA_900626175.2) and Pink Pepper (ASM2916894v1, NCBI GenBank GCA_029168945.1), the previous and current NCBI *C. sativa* reference genomes, respectively, to newly assembled genomes illustrated multiple assembly errors (Figure 4A). BSA data from Toth et al. [9] were re-analyzed using both haplotypes of ‘Panakeia v2.0’ and GVA-H-19-1185-059. The G-statistic peaks in all four new assemblies showed strong agreement (Figure 4A), with slight positional variations in the peak markers among assemblies (Figure 4B). By considering consensus across analyses with all four assemblies as references, the most significant peak region for *Early1* can be narrowed to a ~2 Mb syntenic region on Chr01. This is substantially smaller than the fragmented region observed in CBDRx v2 and Pink Pepper, where numerous small peaks span over 40 Mb (Figure 4A).

Using the data from the BSA, SNPs associated with *Early1* were identified in ‘Panakeia v2.0’ hap1 as well as 22 other *C. sativa* assemblies (Supplementary Table S3). The ‘Panakeia v2.0’ hap1 assembly also has a large ~20 Mb inversion on the same arm of Chr01, which is found in 21 other *C. sativa* assemblies (Supplementary Table S3). The only assembly with both the inversion and the *Early1*-associated SNPs is ‘Panakeia v2.0’ hap1 (Supplementary Table S3).

## 3. Discussion

This study provides new insights into the architecture and composition of the *Cannabis sativa* genome through the analysis of seven high-quality, haplotype-phased assemblies representing individuals with diverse cannabinoid profiles, market classes, and sex chromosomes. These assemblies capture previously uncharacterized diversity, providing a unique opportunity to explore *C. sativa* genomic variation. By presenting these assemblies into the context of other *C. sativa* assemblies, we were able to characterize uniqueness both within diploid individuals, comparing AROH, and among assemblies, using unique *k*-mers. Leveraging genotypes previously used to develop bi-parental mapping populations allowed us to integrate existing linkage map data, enabling detailed estimation of recombination landscapes across the genome. Additionally, we were able to narrow the position of the *Early1* locus by re-analyzing publicly available BSA data with improved assemblies and considering the impact of reference bias. These assemblies will not only contribute to a more comprehensive *C. sativa* pangenome but, through maintaining the associated living individuals and their progeny, will provide essential resources to support future research in *C. sativa* physiology, biochemistry, horticulture, breeding, and many other disciplines.

### 3.1. Priorities for Future C. sativa Genome Sequencing

Despite the recent surge in *C. sativa* genome assemblies, the PanKmer collector’s curve (Figure 1D) and unique *k*-mer analysis (Figure 1A, Supplementary Table S2) suggest that substantial portions of *C. sativa* germplasm remain under-sampled or unsampled entirely. The unique *k*-mer metric is an effective indicator of the extent to which germplasm has been adequately sampled. As sampling increases, haplotypes and their associated *k*-mers are repeatedly captured, resulting in fewer unique *k*-mers in the index. Consequently, when germplasm is well-sampled, there will be fewer unique *k*-mers in any given haplotype.

According to this metric, one could argue that the only well-sampled germplasm is the OCBD breeding program. This makes sense considering the large number of high-quality assemblies derived from this high-cannabinoid breeding program, many of which are closely related [25]. Additionally, the French hemp cultivar ‘Santhica’ appears relatively well-sampled, with numerous assemblies from related individuals or derivatives, including FCS1b, GVA-H-19-1185-059, H3S1b, H3S7b, SAN2, SGVA, and SN1v3, all showing relatively low counts of unique *k*-mers. This cultivar and its derivatives have been a focus of sequencing efforts due to its uncommon CBG-dominant cannabinoid profile (also known as chemotype IV) [50].

Given the vast genetic diversity yet to be sampled, future sequencing efforts should prioritize unsampled populations, particularly those originating from Central, South, and Southeast Asia, the Middle East, and Africa. These regions, especially around hypothesized centers of domestication, are likely to harbor extensive genetic diversity. Additional sampling of East Asian hemp should also be prioritized, as the large numbers of unique *k*-mers from relatively few individuals suggests this germplasm is under-sampled (Figure 1A).

Historical admixture resulting from intentional breeding and movement of *C. sativa* by humans will also contribute to haplotype diversity as more genomes are assembled. Crosses between Asian and European hemp populations are reported to have resulted in cultivars including ‘Carmagnola’, ‘Kompolti’ (KOMPa, KOMPb), and many of their derivatives [51,52,53,54,55]. Genomic studies have also suggested that certain U.S. feral hemp populations are closely related to Italian fiber cultivars like ‘Carmagnola’ [18], while others may share ancestry with East Asian populations [2]. Early 20th-century breeding programs, such as those led by Lyster Dewey, likely contributed to this complex population structure through the development of germplasm pools like ‘Kentucky hemp’ resulting from crosses among Asian, European, and U.S. feral hemp populations [56]. Future sequencing of feral populations may be able to resolve admixed haplotypes that have been maintained over decades of natural selection. Admixture is also evident in the recent development of high-cannabidiol (CBD) germplasm resulting from the introgression of cannabidiolic acid synthase (CBDAS) from European hemp into marijuana germplasm [6]. For example, the high-CBD cultivar ‘BoAx’ (BOAXa, BOAXb) clusters loosely with European hemp in the cladogram (Figure 1A), while also showing similarity to high-cannabinoid genotypes in the heatmap (Supplementary Figure S1), indicative of admixed haplotypes.

### 3.2. Characterizing Homozygosity

Understanding the close relationship between inbreeding and heterosis is fundamental to developing inbred-hybrid breeding systems that dominate many cropping systems [57]. Dioecious species like *C. sativa*, which are typically obligate outcrossers, maintain high levels of heterozygosity and genetic load, resulting in inbreeding depression when plants are self-pollinated, complicating the development of inbred lines [58]. Generating genome assemblies and sequencing diversity panels will help to describe the architecture of genetic load in *C. sativa* while also identifying haplotypes that can be homozygous without deleterious effects. By using alignment-based metrics, like AROH, we can leverage additional information, like structural variants from genome assemblies and long-read sequence data, to more precisely quantify heterozygosity and inform inbreeding strategies. The coverage of AROH in the ‘FL 58’ and GERv1 assemblies suggests that large portions of the genome can be homozygous while maintaining viability.

In this study, the length, coverage, and positions of AROH provide insights into the breeding histories of several lines. For instance, ‘FL 58’ from Sunrise Genetics, an inbred line produced through multiple generations of self-pollination, is expected to have extensive homozygous regions (C. J. Schwartz, personal communication). This is consistent with the large regions of AROH. Similarly, GVA-H-21-1003-002, derived from a small isolated feral population in New York and subjected to further artificial selection and inbreeding, also exhibits long AROH.

### 3.3. Recombination Rates

Recombination rates play a crucial role in plant breeding, particularly in the introgression of desired alleles. In regions with low recombination rates, linkage drag is substantial, requiring multiple generations to break the LD between beneficial and deleterious alleles. Conversely, deleterious alleles in regions of higher recombination are more easily purged [59]. We detected a periphery-biased recombination pattern in *C. sativa*, consistent with the analysis by Laverty et al. [4], and common in organisms with long chromosomes [60,61]. This bias will have substantial implications for breeders accessing beneficial alleles found in the centers of chromosomes while also mitigating linkage drag. In addition to applied plant breeding, variation in recombination rate directly impacts linkage mapping resolution. Much larger population sizes will be necessary to narrow QTL in regions with low recombination rates.

An interesting case of repressed recombination is the short arm of Chr08. As Chr08 is subtelocentric, one hypothesis is that the proximity to the centromere represses recombination on the short arm. However, crossover inhibition explicitly due to the centromere typically extends only a few Mb, as is the case in rice [62], which would not be expected to cover the entire 8–10 Mb of the short arm [60]. Another hypothesis is that structural variants between haplotypes in an F_1_ could locally reduce recombination rates. Interestingly, both ‘FL 58’ and ‘TJ’s CBD’ have large structural variants in that region (Figure 2, Supplementary Figure S2), which likely contribute to the locally repressed recombination. In other populations without structural variants, we would expect greater recombination rates on the short arm of Chr08.

### 3.4. Marker Segregation Impacts Linkage Map Construction

Linkage map construction relies on the predictable segregation patterns expected in structured populations to build genetic maps. Due to this assumption, distorted markers deviating from expected segregation ratios are often purged during map construction, which can leave gaps in genetic maps. This is the case for Chr08 in the linkage maps constructed from the GVA-H-21-1005 family and the combined families (Figure 3). The expected 1:2:1 segregation across Chr08 in the GVA-H-21-1004 map and the large AROH on Chr08 in ‘FL 58’ suggest that the segregation distortion is associated with inheriting one of the haplotypes from ‘TJ’s CBD’. Several factors, including transmission ratio distortion, a recessive lethal locus, or a seed dormancy locus, could result in observed segregation distortion, but more studies will be necessary to identify the specific factor in the parental genome.

Regions lacking segregating markers can also result in gaps during linkage map construction, as we observed on Chr03 and 07 (Figure 3). In F_2_ populations, this could be a result of long stretches of homozygosity in the F_1_ that was self-pollinated. It is possible, for example, that shared haplotypes exist in ‘FL 58’ and ‘TJ’s CBD’ that would always produce monomorphic markers as the regions are truly homozygous in F_1_ progeny. It is also possible that regions may be heterozygous but only have monomorphic markers due to ascertainment bias in the design of marker panels. In the case of the array used to genotype the ‘FL 58’ × ‘TJ’s CBD’ F_2_ populations, which was designed by a company primarily working with high-tetrahydrocannabinol (THC) germplasm, markers that more commonly segregate in high-CBD germplasm and not high-THC germplasm may have been excluded.

### 3.5. Reference Bias Impacts Trait Mapping Using BSA

Using high-quality genome assemblies for alignment is critical for high-resolution trait mapping, as assembly errors introduce significant noise that complicates analyses. When re-analyzing BSA data for the *Early1* flowering locus using several assemblies as references, assembly errors—particularly in CBDRx v2 and Pink Pepper—exemplify extreme cases of reference-specific results. By using higher-quality assemblies and integrating data across multiple references, a consensus was reached that substantially narrows the *Early1* locus. This consensus approach not only enhances the precision of trait mapping but also mitigates alignment biases and errors inherent in single-reference analyses. Such multi-reference analyses, while more complex, provide a more robust foundation for identifying candidate genes associated with important traits in *C. sativa*.

Even in well assembled genomes, structural variants can introduce variation that influences alignment results. This is evident on Chr01 of ‘Panakeia v2.0’ hap1 as the large ~20 Mb inversion shifts part of the region above the BSA significance threshold to the end of the chromosome (Figure 4A). This pattern suggests that, despite ‘Panakeia v2.0’ hap1 having the SNPs associated with *Early1*, the ‘Umpqua’ population sequenced by Toth et al. [9] did not have the inversion linked to *Early1.* Interestingly, ‘Panakeia v2.0’ hap1 is the only haplotype from a phased chromosome-level assembly that has both the *Early1* associated SNPs and the ~20 Mb inversion (Supplementary Table S3). This could be a result of a recombination event between the locus and the inversion, or potentially an error in phasing some of the assemblies. In either case, specific consideration for structural variants, which could be causative or linked to causative loci, is critical when selecting assemblies for alignment.

### 3.6. Associated Phenotypes and Loci of Interest

Genome assemblies in isolation are valuable, but they are significantly more valuable when they are associated with meaningful phenotypic data and exponentially more valuable when the sequenced individual is alive and available for other researchers to study. All seven genotypes sequenced in this manuscript are being maintained through cutting propagation, and several have been contributed to the U.S. NPGS hemp collection. Additionally, there are numerous publications associated with genotypes (Table 2).

Several loci with alleles known to impact disease resistance, flowering time, and secondary metabolism are present in this set of genomes. ‘FL 58’ is homozygous for a mutation in *CsMLO1*, a powdery mildew susceptibility gene on Chr01, and several other loci that contribute to powdery mildew resistance [10]. GVA-H-21-1003-002 is homozygous for the flowering time locus *Autoflower2* [12]. Finally, GVA-H-19-1067-001 has a consistently high proportion of CBC(A) in its cannabinoid profile [44] and has a sequence matching the “expressed” cannabichromenic acid synthase (CBCAS) described in the patent application from Canopy Growth Corporation [63].

### 3.7. Future Directions

This study provides substantial data toward the development of comprehensive pangenomic resources that capture the complete genetic diversity of *C. sativa* and mitigate reference bias. These efforts will deepen our understanding of sex chromosomes, structural variation, homozygosity, and population structure that will provide insight into local adaptation and phenotypic diversity. Future studies should also explore recombination rate variation among diverse populations, as well as artificial manipulation of recombination rates, which could accelerate breeding by reducing linkage drag associated with trait introgression and recurrent selection. As high-throughput phenotyping technologies evolve, integrating genome assemblies with associated phenotypic data from living and publicly available germplasm will support foundational and applied research on one of humanity’s oldest sources of food, fiber, and medicine.

## 4. Materials and Methods

### 4.1. Plant Materials, DNA Isolation, and Sample Sequencing

Seven cutting-propagated hemp genotypes from various market classes and diversity groups were selected from the Cornell University hemp research group’s collection to generate high-quality genome assemblies (Table 2). Individual plants representing each of these genotypes that had been previously maintained in a greenhouse under >16 h of light were moved into complete darkness for five days. Several grams of etiolated shoot tips were collected from each genotype, flash-frozen, and stored at −80 °C High-molecular-weight DNA was isolated from shoot tips using the protocol described by Schalamun and Schwessinger [64]. The material for GVA-H-19-1185-059 was sequenced at Oregon CBD (Independence, OR, USA), and the remainder of the samples were sequenced at the University of Wisconsin–Madison Biotechnology Center. PacBio HiFi data were generated for all genotypes using a PacBio Sequel II. Frozen tissues of all seven genotypes were sent to the HudsonAlpha Institute for Biotechnology (Huntsville, AL, USA) to generate Dovetail^®^ Omni-C^®^ libraries. Libraries were constructed using standard protocols (Dovetail Omni-C Kit Catalog #21005, Cantata Bio, Scotts Valley, CA, USA). One gram of flash-frozen leaf tissue was ground, and after nuclei isolation, the pellet was resuspended in 1X PBS, aliquoted into 3 tubes, and centrifuged at 6000 rpm for 5 min. The supernatant was then discarded, and the nuclei pellets were flash-frozen for storage at −80 degrees. One aliquoted nuclei pellet was then processed using the Dovetail^®^ Omni-C^®^ protocol V2. Libraries were run on an Illumina NovaSeq 6000 PE150.

### 4.2. Genome Assembly and Annotation

For each genotype, HiFi and Omni-C^®^ sequence data were used to generate contig-level assemblies. First, Omni-C^®^ data were trimmed and filtered using fastp [65], and then both the HiFi and Omni-C^®^ data were used as inputs for hifiasm [66]. To identify and phase the sex chromosomes in the two XY assemblies, we employed the method described by Carey et al. [26]. Briefly, male-specific *k*-mers (Y-mers) were mapped to the contig-level assemblies, and a combined assembly using contigs from both haplotypes was constructed to verify that Y- and X-tigs were appropriately phased into separate haplotypes. For the five XX assemblies, we did not perform additional manual phasing prior to scaffolding. Contig-level assemblies were screened for contaminants using FCS-GX [67]. To scaffold the contig-level assemblies, the Omni-C^®^ reads were aligned to their respective contigs for each of the 14 haplotypes using the Dovetail^®^ Omni-C^®^ pipeline [68]. These alignments and the contig-level assemblies were used as inputs for YaHS [69] to generate scaffolded assemblies. Finally, scaffolded assemblies were manually curated based on contact maps generated and visualized using Juicer and Juicebox [70] to generate chromosome-level assemblies. Chromosomes were ordered and oriented, adhering to the CBDRx v2 genome assembly conventions [6], based on alignment to the ‘Carmagnola’ assembly [26] using D-GENIES [71] implementing minimap2 [49].

Structural gene annotations were predicted ab initio using Helixer v0.3.3 [38]. Protein sequences were extracted using GffRead [72] and used to generate functional gene annotations with eggNOG-mapper v2.1.12 [73]. To assess assembly quality, BUSCO v5.7.1 [74] using the embryophyta_odb10 database was run on the final manually curated assemblies as well as the predicted proteins from Helixer.

PAR-SDR boundaries for the two-phased XY assemblies were identified by mapping Y-mers from Carey et al. [26] to both X/Y haplotypes, then analyzing gene trees of syntenic orthologs conserved in all eight available ChrY assemblies for those spanning the putative PAR-SDR boundary (i.e., the ChrY region with decreased Y-mer density). Y-mers were mapped to all four X/Y haplotypes using BWA mem v.0.7.17, requiring perfect alignments and multimapping up to 10 times. Orthologs were identified using OrthoFinder v2.5.4 with the multiple sequence alignment option. Blastn (BLAST+ v.2.14.1) and bedtools (v.2.31.0) getfasta were used to identify and extract nucleotide sequences from each X/Y assembly for 10 conserved orthologs spanning the putative PAR-SDR boundary. Each gene tree was estimated based on a MAFFT v.7.505-generated multiple sequence alignment, using the options --*localpair* and --*maxiterate 1000*, and maximum likelihood tree inference with IQ-TREE v.1.6.12 with the options *-MFP* and –*bb 1000.* The SDR starting position on the Y was defined 10 bp upstream of the first putatively SDR-linked gene model.

### 4.3. Generation of a PanKmer Index and Identification of Unique k-mers

PanKmer [39] was used to generate a *k*-mer-based pangenomic index for all of the assemblies available from NCBI, Lynch et al. [25], Carey et al. [26], and those presented in this study. Subsequently, custom bash and python scripts were used to identify unique *k*-mers from the index. For the assemblies presented in this study, unique *k*-mers were aligned back to the chromosome-level assemblies using BWA-MEM [75] to identify regions with greater densities of unique *k*-mers suggesting unique haplotypes that are not present in other assemblies included in the index.

To generate the hierarchical clustering tree, a second index was generated with only the phased assemblies. The index was initially constructed using sequences from all chromosomes of both chromosome-level and contig-level assemblies, but this resulted in clustering of haplotypes with Y chromosomes (Supplementary Figure S3). To avoid bias in clustering due to the sex chromosomes, the index was reconstructed using only autosomal sequence data for the 100 haplotypes from the 50 phased chromosome-level assemblies. To reduce memory and run time, an incomplete Q30 index was generated. The PanKmer adj-matrix and tree functions were then used to generate a hierarchical clustering and heatmap of genomes using Jaccard similarity scores.

### 4.4. Pairwise Alignment of Haplotypes

To visualize syntenic regions of homologous chromosomes, pairwise alignment of the two haplotypes for each of the diploid phased chromosome-level assemblies (n = 50) was performed using D-GENIES [71] implementing minimap2 [49]. The resulting PAF files were processed with a custom R [76] script to merge artificial breaks in alignments that were frequently introduced when aligned regions exceeded 10 Mb. A primary pairwise alignment between haplotypes was classified as an aligned run of homozygosity (AROH) when it was greater than 50 kb in length and had an estimated per-base sequence divergence of less than 0.01.

### 4.5. Recombination Frequency Estimation

An improved set of linkage maps was constructed from the ‘FL 58’ × ‘TJ’s CBD’ bi-parental F_2_ mapping population data [10], and genetic map positions were used to estimate recombination frequency across the genome. The R packages ASMap v1.0-8 [77] and qtl v1.70 [78] were used to construct the linkage maps. Biallelic F_2_ marker data from the Illumina array were coded such that allele A was contributed by ‘FL 58’ and allele B was contributed by ‘TJ’s CBD’. A total of three linkage maps were constructed: one for each F_2_ population, GVA-H-21-1004 and GVA-H-21-1005, and one using marker data of both populations combined. Markers that had significant segregation distortion (threshold = $1\times 10^{-5}$ for GVA-H-21-1004; $1\times 10^{-17}$ for GVA-H-21-1005; $1\times 10^{-5}$ for both populations), had a significant proportion of missing data (threshold > 0.35), and were in complete linkage disequilibrium with other markers were removed using the *pullCross* function. The *mstmap* function was used for genetic distance calculation, marker ordering, and linkage group clustering, specifying the following *p*-value thresholds: $1\times 10^{-50},\;1\times 10^{-45},\;and\;1\times 10^{-80}$ for GVA-H-21-1004, GVA-H-21-1005, and both populations, respectively. Small linkage groups that did not represent an entire chromosome were merged with linkage groups whose markers had low estimated pairwise recombination and high LOD linkage. The calculation of double crossovers per marker was determined by the *profileMark* function. Through an iterative process, markers in the GVA-H-21-1004 and GVA-H-21-1005 populations that exceeded two double crossovers were removed, while markers with no more than one double crossover were dropped for the linkage map using the data of both populations. Linkage groups with less than or equal to 21, 20, and 10 markers were discarded in the maps of GVA-H-21-1004, GVA-H-21-1005, and both populations combined, respectively. Final linkage groups were assigned chromosome numbering and orientation using the *flip.order* function based on marker positions on the ‘FL 58’ hap1 assembly. Individual markers that excessively deviated from their expected physical location given their genetic location were dropped.

To estimate recombination rates, array probe sequences for markers in any of the three linkage maps were aligned to the ‘FL 58’ hap2 assembly using blastn [79]. Alignment of genetic and physical maps was visualized by plotting the coordinates of the markers in the genetic map against their positions in the physical map. Three regions that were duplicated and inverted on Chr03, 06, and 09 were manually removed from the GVA-H-21-1005 genetic map. LOESS models were fit for each chromosome using a span of 0.25, and the absolute value of the numerical derivative between successive fitted values was calculated to estimate the recombination rate at each marker position. MAF was calculated at the family level for all markers that were included in any of the three linkage maps.

### 4.6. Early1 BSA Aligning to Various Genome Assemblies

The *Early1* BSA data from Toth et al. [9] (NCBI BioProject PRJNA856865) were downloaded and filtered with fastp [65] using default settings. Then, NVIDIA Clara ParaBricks v4.3.1-1 fq2bam, a GPU-accelerated wrapper of BWA-MEM [75], was used to assign read groups, reference index, and align the reads to each of the six reference assemblies: CBDRx v2, Pink Pepper, both haplotypes of ‘Panakeia v2.0’, and both haplotypes of GVA-H-19-1185-059. Following alignment, Clara Parabricks haplotypecaller was used to call variants and generate GVCF files for each read group. GVCF files were combined, and variants were jointly called using CombineGVCFs and GenotypeGVCFs from GATK [80]. The VCF file was filtered to a minimum depth of 10 reads and converted to table format using VCFtools [81]. The table was converted to a CSV, and PyBSASeq [82] using default parameters for a backcross population was used to conduct the BSA.

The output files from PyBSASeq were used to identify a consensus peak interval spanning ~2 Mb on Chr01. To identify molecular markers associated with the presence of *Early1*, 20 SNP sites were identified across the peak interval. SNPs were identified based on concurrence with expected allele frequencies and sufficient read depth. SNPs and flanking sequences were aligned to 105 chromosome-level assemblies using Persephone^®^ [83]. Comparing the calls for the 20 SNPs, we narrowed the list to five SNPs that produced consistent calls among all genomes and are likely linked to the causative variant for *Early1*. These five SNPs were used to identify whether or not each of the 100 phased chromosome-level assemblies is likely to have *Early1*.

All figures were generated in R [76] using base functionality or ggplot2 [84].

## Figures and Tables

**Table 1:** Summary of several population structure analyses conducted on *C. sativa.*

Publication	N Samples	N Groups	High-Cannabinoid Group(s)	European Hemp Group(s)	Asian Hemp Group	Other Group(s)
Sawler et al. [17]	124	2	Marijuana	Hemp	n/a	n/a
Soorni et al. [30]	209	4	Marijuana	Hemp CGN/IPK	n/a	Iran
Lynch et al. [31]	340	3	BLDT NLDT	Hemp	n/a	n/a
Grassa et al. [6]	367	3	Marijuana	Hemp	n/a	Naturalized
Carlson et al. [18]	190	7	T1/R4; Cherry West Coast BaOx/Otto II	Grain/Dual Fiber/Feral	Chinese	n/a
Ren et al. [2]	110	4	Drug-type Drug-type Feral	Hemp-type	n/a	Basal Cannabis
Woods et al. [19]	190	4	Marijuana	European	Asian	U.S. Feral
Lynch et al. [25]	193	5	Drug-type ERB and EH23b Drug-type HO40 and EH23a	European Hemp and Feral	Asian Hemp	Wild Tibet

## Data Availability

All of the genome assemblies and raw sequence data are available as a part of NBCI BioProject PRJNA1193891. Additional code and intermediate data analyses can be found at https://github.com/GMStack/2024_Hemp_Genomes (Last accessed 28 January 2025) or from the authors upon reasonable request.

## Supplementary Materials

The following supporting information can be downloaded at: https://www.mdpi.com/article/10.3390/ijms26031165/s1.

## References

[1] Kovalchuk I., Pellino M., Rigault P., van Velzen R., Ebersbach J., Ashnest J.R., Mau M., Schranz M.E., Alcorn J., Laprairie R.B. (2020). The Genomics of *Cannabis* and Its Close Relatives. Annu. Rev. Plant Biol..

[2] Ren G., Zhang X., Li Y., Ridout K., Serrano-Serrano M.L., Yang Y., Liu A., Ravikanth G., Nawaz M.A., Mumtaz A.S. (2021). Large-Scale Whole-Genome Resequencing Unravels the Domestication History of *Cannabis sativa*. Sci. Adv..

[3] Van Bakel H., Stout J.M., Cote A.G., Tallon C.M., Sharpe A.G., Hughes T.R., Page J.E. (2011). The Draft Genome and Transcriptome of *Cannabis sativa*. Genome Biol..

[4] Laverty K.U., Stout J.M., Sullivan M.J., Shah H., Gill N., Holbrook L., Deikus G., Sebra R., Hughes T.R., Page J.E. (2019). A Physical and Genetic Map of *Cannabis sativa* Identifies Extensive Rearrangements at the THC/CBD Acid Synthase Loci. Genome Res..

[5] Gao S., Wang B., Xie S., Xu X., Zhang J., Pei L., Yu Y., Yang W., Zhang Y. (2020). A High-Quality Reference Genome of Wild *Cannabis sativa*. Hortic. Res..

[6] Grassa C.J., Weiblen G.D., Wenger J.P., Dabney C., Poplawski S.G., Timothy Motley S., Michael T.P., Schwartz C.J. (2021). A New Cannabis Genome Assembly Associates Elevated Cannabidiol (CBD) with Hemp Introgressed into Marijuana. New Phytol..

[7] Mihalyov P.D., Garfinkel A.R. (2021). Discovery and Genetic Mapping of PM1, a Powdery Mildew Resistance Gene in *Cannabis sativa* L.. Front. Agron..

[8] Woods P., Campbell B.J., Nicodemus T.J., Cahoon E.B., Mullen J.L., McKay J.K. (2021). Quantitative Trait Loci Controlling Agronomic and Biochemical Traits in *Cannabis sativa*. Genetics.

[9] Toth J.A., Stack G.M., Carlson C.H., Smart L.B. (2022). Identification and Mapping of Major-Effect Flowering Time Loci Autoflower1 and Early1 in *Cannabis sativa* L.. Front. Plant Sci..

[10] Stack G.M., Cala A.R., Quade M.A., Toth J.A., Monserrate L.A., Wilkerson D.G., Carlson C.H., Mamerto A., Michael T.P., Crawford S. (2023). Genetic Mapping, Identification, and Characterization of a Candidate Susceptibility Gene for Powdery Mildew in *Cannabis sativa* L.. Mol. Plant. Microbe. Interact..

[11] Steel L., Welling M., Ristevski N., Johnson K., Gendall A. (2023). Comparative Genomics of Flowering Behavior in *Cannabis sativa*. Front. Plant Sci..

[12] Dowling C.A., Shi J., Toth J.A., Quade M.A., Smart L.B., McCabe P.F., Schilling S., Melzer R. (2024). A Flowering Locus T Ortholog Is Associated with Photoperiod-Insensitive Flowering in Hemp (*Cannabis sativa* L.). Plant J..

[13] Petit J., Salentijn E.M.J., Paulo M.-J., Denneboom C., van Loo E.N., Trindade L.M. (2020). Elucidating the Genetic Architecture of Fiber Quality in Hemp (*Cannabis sativa* L.) Using a Genome-Wide Association Study. Front. Genet..

[14] Petit J., Salentijn E.M.J., Paulo M.-J., Denneboom C., Trindade L.M. (2020). Genetic Architecture of Flowering Time and Sex Determination in Hemp (*Cannabis sativa* L.): A Genome-Wide Association Study. Front. Plant Sci..

[15] Welling M.T., Liu L., Kretzschmar T., Mauleon R. (2020). An Extreme-Phenotype Genome-wide Association Study Identifies Candidate Cannabinoid Pathway Genes in Cannabis. Sci. Rep..

[16] De Ronne M., Lapierre É., Torkamaneh D. (2024). Genetic Insights into Agronomic and Morphological Traits of Drug-Type Cannabis Revealed by Genome-Wide Association Studies. Sci. Rep..

[17] Sawler J., Stout J.M., Gardner K.M., Hudson D., Vidmar J., Butler L., Page J.E., Myles S. (2015). The Genetic Structure of Marijuana and Hemp. PLoS ONE.

[18] Carlson C.H., Stack G.M., Jiang Y., Taşkıran B., Cala A.R., Toth J.A., Philippe G., Rose J.K.C., Smart C.D., Smart L.B. (2021). Morphometric Relationships and Their Contribution to Biomass and Cannabinoid Yield in Hybrids of Hemp (*Cannabis sativa*). J. Exp. Bot..

[19] Woods P., Price N., Matthews P., McKay J.K. (2022). Genome-Wide Polymorphism and Genic Selection in Feral and Domesticated Lineages of *Cannabis sativa*. G3.

[20] Allen K.D., McKernan K., Pauli C., Roe J., Torres A., Gaudino R. (2019). Genomic Characterization of the Complete Terpene Synthase Gene Family from *Cannabis sativa*. PLoS ONE.

[21] Pépin N., Hebert F.O., Joly D.L. (2021). Genome-Wide Characterization of the MLO Gene Family in *Cannabis sativa* Reveals Two Genes as Strong Candidates for Powdery Mildew Susceptibility. Front. Plant Sci..

[22] Borrego E.J., Robertson M., Taylor J., Schultzhaus Z., Espinoza E.M. (2023). Oxylipin Biosynthetic Gene Families of *Cannabis sativa*. PLoS ONE.

[23] Hurgobin B., Edwards D. (2017). SNP Discovery Using a Pangenome: Has the Single Reference Approach Become Obsolete?. Biology.

[24] Bradbury P.J., Casstevens T., Jensen S.E., Johnson L.C., Miller Z.R., Monier B., Romay M.C., Song B., Buckler E.S. (2022). The Practical Haplotype Graph, a Platform for Storing and Using Pangenomes for Imputation. Bioinformatics.

[25] Lynch R.C., Padgitt-Cobb L.K., Garfinkel A.R., Knaus B.J., Hartwick N.T., Allsing N., Aylward A., Mamerto A., Kitony J.K., Colt K. (2024). Domesticated Cannabinoid Synthases amid a Wild Mosaic Cannabis Pangenome. bioRxiv.

[26] Carey S.B., Bentz P.C., Lovell J.T., Akozbek L.M., Havill J.S., Padgitt-Cobb L., Lynch R.C., Allsing N., Osmanski A., Easterling K.A. (2024). The Evolution of Heteromorphic Sex Chromosomes in Plants. bioRxiv.

[27] Hirata K. (1927). Sex Determination in Hemp (*Cannabis sativa* L.). J. Genet..

[28] Small E., Cronquist A. (1976). A Practical and Natural Taxonomy for *Cannabis*. Taxon.

[29] McPartland J.M., Small E. (2020). A Classification of Endangered High-THC Cannabis (*Cannabis sativa* Subsp.. Indica) Domesticates and Their Wild Relatives. PhytoKeys.

[30] Soorni A., Fatahi R., Haak D.C., Salami S.A., Bombarely A. (2017). Assessment of Genetic Diversity and Population Structure in Iranian Cannabis Germplasm. Sci. Rep..

[31] Lynch R.C., Vergara D., Tittes S., White K., Schwartz C.J., Gibbs M.J., Ruthenburg T.C., deCesare K., Land D.P., Kane N.C. (2016). Genomic and Chemical Diversity in *Cannabis*. Crit. Rev. Plant Sci..

[32] Brandt D.Y.C., Aguiar V.R.C., Bitarello B.D., Nunes K., Goudet J., Meyer D. (2015). Mapping Bias Overestimates Reference Allele Frequencies at the HLA Genes in the 1000 Genomes Project Phase I Data. G3.

[33] Günther T., Nettelblad C. (2019). The Presence and Impact of Reference Bias on Population Genomic Studies of Prehistoric Human Populations. PLoS Genet..

[34] Hickey G., Monlong J., Ebler J., Novak A.M., Eizenga J.M., Gao Y., Marschall T., Li H., Paten B., Human Pangenome Reference Consortium (2024). Pangenome Graph Construction from Genome Alignments with Minigraph-Cactus. Nat. Biotechnol..

[35] Valiente-Mullor C., Beamud B., Ansari I., Francés-Cuesta C., García-González N., Mejía L., Ruiz-Hueso P., González-Candelas F. (2021). One Is Not Enough: On the Effects of Reference Genome for the Mapping and Subsequent Analyses of Short-Reads. PLoS Comput. Biol..

[36] Epstein R., Sajai N., Zelkowski M., Zhou A., Robbins K.R., Pawlowski W.P. (2023). Exploring Impact of Recombination Landscapes on Breeding Outcomes. Proc. Natl. Acad. Sci. USA.

[37] Taagen E., Bogdanove A.J., Sorrells M.E. (2020). Counting on Crossovers: Controlled Recombination for Plant Breeding. Trends Plant Sci..

[38] Holst F., Bolger A., Günther C., Maß J., Triesch S., Kindel F., Kiel N., Saadat N., Ebenhöh O., Usadel B. (2023). Helixer–de Novo Prediction of Primary Eukaryotic Gene Models Combining Deep Learning and a Hidden Markov Model. bioRxiv.

[39] Aylward A.J., Petrus S., Mamerto A., Hartwick N.T., Michael T.P. (2023). PanKmer: K-Mer-Based and Reference-Free Pangenome Analysis. Bioinformatics.

[40] Mandolino G., Carboni A. (2004). Potential of Marker-Assisted Selection in Hemp Genetic Improvement. Euphytica.

[41] Lu Y., Young S., Linder E., Whipker B., Suchoff D. (2021). Hyperspectral Imaging With Machine Learning to Differentiate Cultivars, Growth Stages, Flowers, and Leaves of Industrial Hemp (*Cannabis sativa* L.). Front. Plant Sci..

[42] Stack G.M., Toth J.A., Carlson C.H., Cala A.R., Marrero-González M.I., Wilk R.L., Gentner D.R., Crawford J.L., Philippe G., Rose J.K.C. (2021). Season-long Characterization of High-cannabinoid Hemp (*Cannabis sativa* L.) Reveals Variation in Cannabinoid Accumulation, Flowering Time, and Disease Resistance. Glob. Change Biol. Bioenergy.

[43] Smart L.B., Toth J.A., Stack G.M., Monserrate L.A., Smart C.D., Goldman I. (2022). Breeding of Hemp (*Cannabis sativa*). Plant Breeding Reviews.

[44] Stack G.M., Carlson C.H., Toth J.A., Philippe G., Crawford J.L., Hansen J.L., Viands D.R., Rose J.K.C., Smart L.B. (2023). Correlations among Morphological and Biochemical Traits in High-Cannabidiol Hemp (*Cannabis sativa* L.). Plant Direct..

[45] Weldon W.A., Ullrich M.R., Smart L.B., Smart C.D., Gadoury D.M. (2020). Cross-Infectivity of Powdery Mildew Isolates Originating from Hemp (*Cannabis sativa*) and Japanese Hop (*Humulus japonicus*) in New York. Plant Health Prog..

[46] Ahmed B., Smart L.B., Hijri M. (2021). Microbiome of Field Grown Hemp Reveals Potential Microbial Interactions With Root and Rhizosphere Soil. Front. Microbiol..

[47] Toth J.A., Smart L.B., Smart C.D., Stack G.M., Carlson C.H., Philippe G., Rose J.K.C. (2021). Limited Effect of Environmental Stress on Cannabinoid Profiles in High-cannabidiol Hemp (*Cannabis sativa* L.). Glob. Change Biol. Bioenergy.

[48] Stephen C., Zayas V.A., Galic A., Bridgen M.P. (2023). Micropropagation of Hemp (*Cannabis sativa* L.). HortScience.

[49] Li H. (2018). Minimap2: Pairwise Alignment for Nucleotide Sequences. Bioinformatics.

[50] Fournier G., Beherec O., Bertucelli S. (2004). Santhica 23 et 27: Deux variétés de chanvre (*Cannabis sativa* L.) sans Δ-9-THC. Ann. Toxicol. Anal..

[51] Bócsa I. (1994). Interview Professor Dr. Iván Bócsa, the Breeder of Kompolti Hemp. J. Int. Hemp Assoc..

[52] de Meijer E. (1995). Fibre Hemp Cultivars: A Survey of Origin, Ancestry, Availability and Brief Agronomic Characteristics. J. Int. Hemp Assoc..

[53] Ranalli P. (2004). Current Status and Future Scenarios of Hemp Breeding. Euphytica.

[54] Salentijn E.M.J., Zhang Q., Amaducci S., Yang M., Trindade L.M. (2015). New Developments in Fiber Hemp (*Cannabis sativa* L.) Breeding. Ind. Crops Prod..

[55] Clarke R.C., Merlin M.D. (2016). Cannabis Domestication, Breeding History, Present-Day Genetic Diversity, and Future Prospects. CRC Crit. Rev. Plant Sci..

[56] Dewey L. (1928). Hemp Varieties of Improved Type Are Result of Selection. What ’s New in Agriculture. Yearbook of the United States Department of Agriculture—1927.

[57] Charlesworth D., Willis J.H. (2009). The Genetics of Inbreeding Depression. Nat. Rev. Genet..

[58] Crnokrak P., Barrett S.C.H. (2002). Perspective: Purging the Genetic Load: A Review of the Experimental Evidence. Evolution.

[59] Chun S., Fay J.C. (2011). Evidence for Hitchhiking of Deleterious Mutations within the Human Genome. PLoS Genet..

[60] Haenel Q., Laurentino T.G., Roesti M., Berner D. (2018). Meta-Analysis of Chromosome-Scale Crossover Rate Variation in Eukaryotes and Its Significance to Evolutionary Genomics. Mol. Ecol..

[61] Brazier T., Glémin S. (2022). Diversity and Determinants of Recombination Landscapes in Flowering Plants. PLoS Genet..

[62] Yan H., Jin W., Nagaki K., Tian S., Ouyang S., Buell C.R., Talbert P.B., Henikoff S., Jiang J. (2005). Transcription and Histone Modifications in the Recombination-Free Region Spanning a Rice Centromere. Plant Cell.

[63] Fowler D.K. (2022). Cannabis Plant with Increased Cannabichromenic Acid. U.S. Patent.

[64] Schalamun M., Schwessinger B. (2017). High Molecular Weight GDNA Extraction after Mayjonade et al. Optimised for Eucalyptus for Nanopore Sequencing. Mol. Ecol. Resour..

[65] Chen S., Zhou Y., Chen Y., Gu J. (2018). Fastp: An Ultra-Fast All-in-One FASTQ Preprocessor. Bioinformatics.

[66] Cheng H., Concepcion G.T., Feng X., Zhang H., Li H. (2021). Haplotype-Resolved de Novo Assembly Using Phased Assembly Graphs with Hifiasm. Nat. Methods.

[67] Astashyn A., Tvedte E.S., Sweeney D., Sapojnikov V., Bouk N., Joukov V., Mozes E., Strope P.K., Sylla P.M., Wagner L. (2024). Rapid and Sensitive Detection of Genome Contamination at Scale with FCS-GX. Genome Biol..

[68] Dovetail Genomics Dovetail Omni-C 0.1 Documentation. https://omni-c.readthedocs.io/en/latest/index.html.

[69] Zhou C., McCarthy S.A., Durbin R. (2023). YaHS: Yet Another Hi-C Scaffolding Tool. Bioinformatics.

[70] Durand N.C., Shamim M.S., Machol I., Rao S.S.P., Huntley M.H., Lander E.S., Aiden E.L. (2016). Juicer Provides a One-Click System for Analyzing Loop-Resolution Hi-C Experiments. Cell Syst..

[71] Cabanettes F., Klopp C. (2018). D-GENIES: Dot Plot Large Genomes in an Interactive, Efficient and Simple Way. PeerJ.

[72] Pertea G., Pertea M. (2020). GFF Utilities: GffRead and GffCompare. F1000Res..

[73] Cantalapiedra C.P., Hernández-Plaza A., Letunic I., Bork P., Huerta-Cepas J. (2021). EggNOG-Mapper v2: Functional Annotation, Orthology Assignments, and Domain Prediction at the Metagenomic Scale. Mol. Biol. Evol..

[74] Manni M., Berkeley M.R., Seppey M., Simão F.A., Zdobnov E.M. (2021). BUSCO Update: Novel and Streamlined Workflows along with Broader and Deeper Phylogenetic Coverage for Scoring of Eukaryotic, Prokaryotic, and Viral Genomes. Mol. Biol. Evol..

[75] Li H. (2013). Aligning Sequence Reads, Clone Sequences and Assembly Contigs with BWA-MEM. arXiv.

[76] R Core Team (2024). R: A Language and Environment for Statistical Computing.

[77] Taylor J., Butler D. (2017). R Package ASMap: Efficient Genetic Linkage Map Construction and Diagnosis. J. Stat. Softw..

[78] Broman K.W., Wu H., Sen S., Churchill G.A. (2003). R/Qtl: QTL Mapping in Experimental Crosses. Bioinformatics.

[79] Camacho C., Coulouris G., Avagyan V., Ma N., Papadopoulos J., Bealer K., Madden T.L. (2009). BLAST+: Architecture and Applications. BMC Bioinform..

[80] Van der Auwera G.A., Carneiro M.O., Hartl C., Poplin R., Del Angel G., Levy-Moonshine A., Jordan T., Shakir K., Roazen D., Thibault J. (2013). From FastQ Data to High Confidence Variant Calls: The Genome Analysis Toolkit Best Practices Pipeline. Curr. Protoc. Bioinform..

[81] Danecek P., Auton A., Abecasis G., Albers C.A., Banks E., DePristo M.A., Handsaker R.E., Lunter G., Marth G.T., Sherry S.T. (2011). The Variant Call Format and VCFtools. Bioinformatics.

[82] Zhang J., Panthee D.R. (2020). PyBSASeq: A Simple and Effective Algorithm for Bulked Segregant Analysis with Whole-Genome Sequencing Data. BMC Bioinform..

[83] Persephone Software L.L.C. (2024). Persephone^®^ Genome Browser.

[84] Wickham H. (2016). ggplot2: Elegant Graphics for Data Analysis.

