# Supplementary material for: Comparison of Recombination Rate, Reference Bias, and Unique Pangenomic Haplotypes in Cannabis sativa Using Seven De Novo Genome Assemblies
Source: Int J Mol Sci. 2025 Jan 29;26(3):1165. doi: 10.3390/ijms26031165 (PMC11818205; doi:10.3390/ijms26031165)
Supplement: Supplementary file 1 [file ijms-26-01165-s001.zip › 2024_11_12_Stack_Cannabis_Genomes_IJMS_Supp_Figs.pdf]

Supplementary Figures for "Comparison of recombination rate, reference bias, and novel pangenomic haplotypes across *Cannabis sativa* using seven *de novo* genome assemblies" by Stack et al.

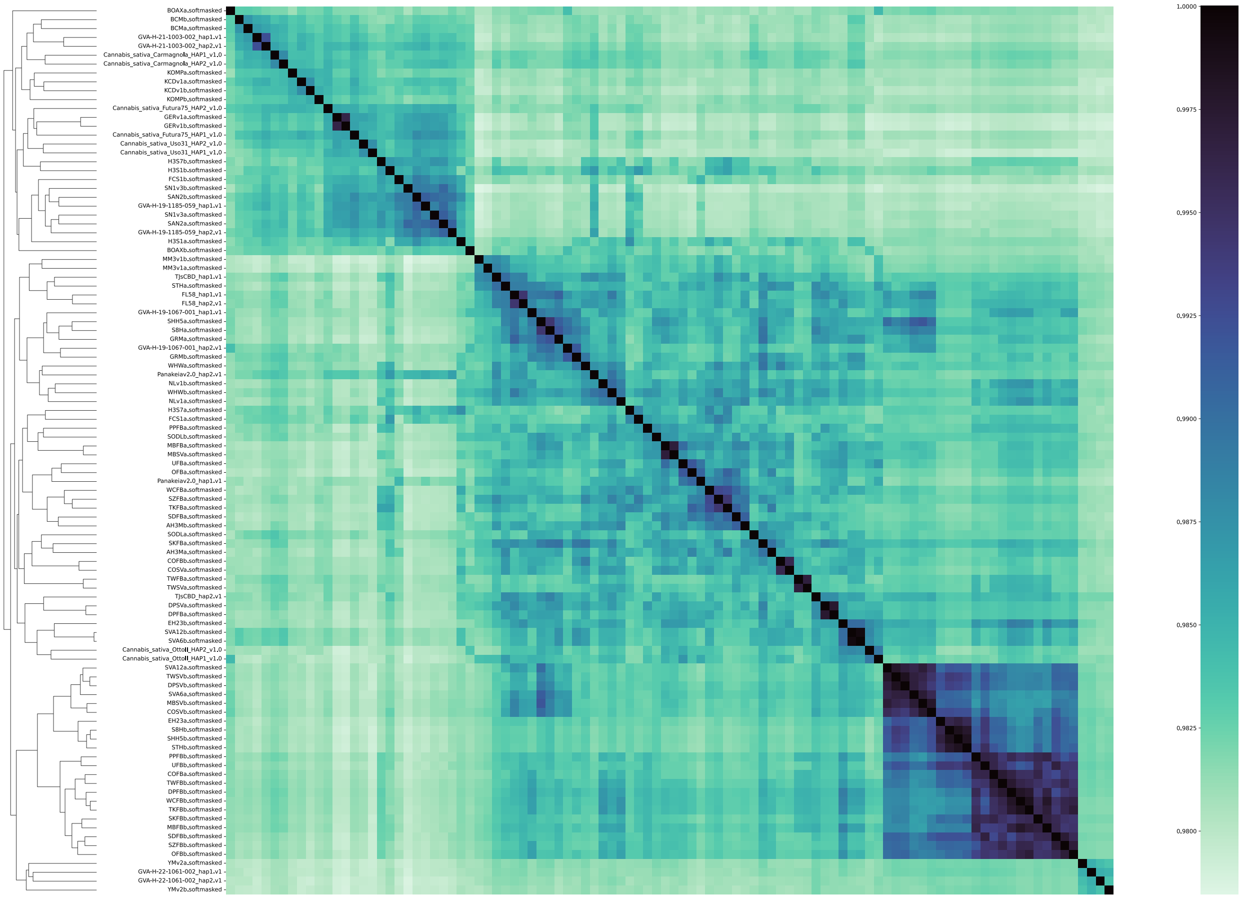

Supplementary Figure S1. Heatmap clustered by Jaccard similarity scores of 100 haplotypes from 50 chromosome-level phased *C. sativa* assemblies based on an autosomal 31-mer PanKmer index.

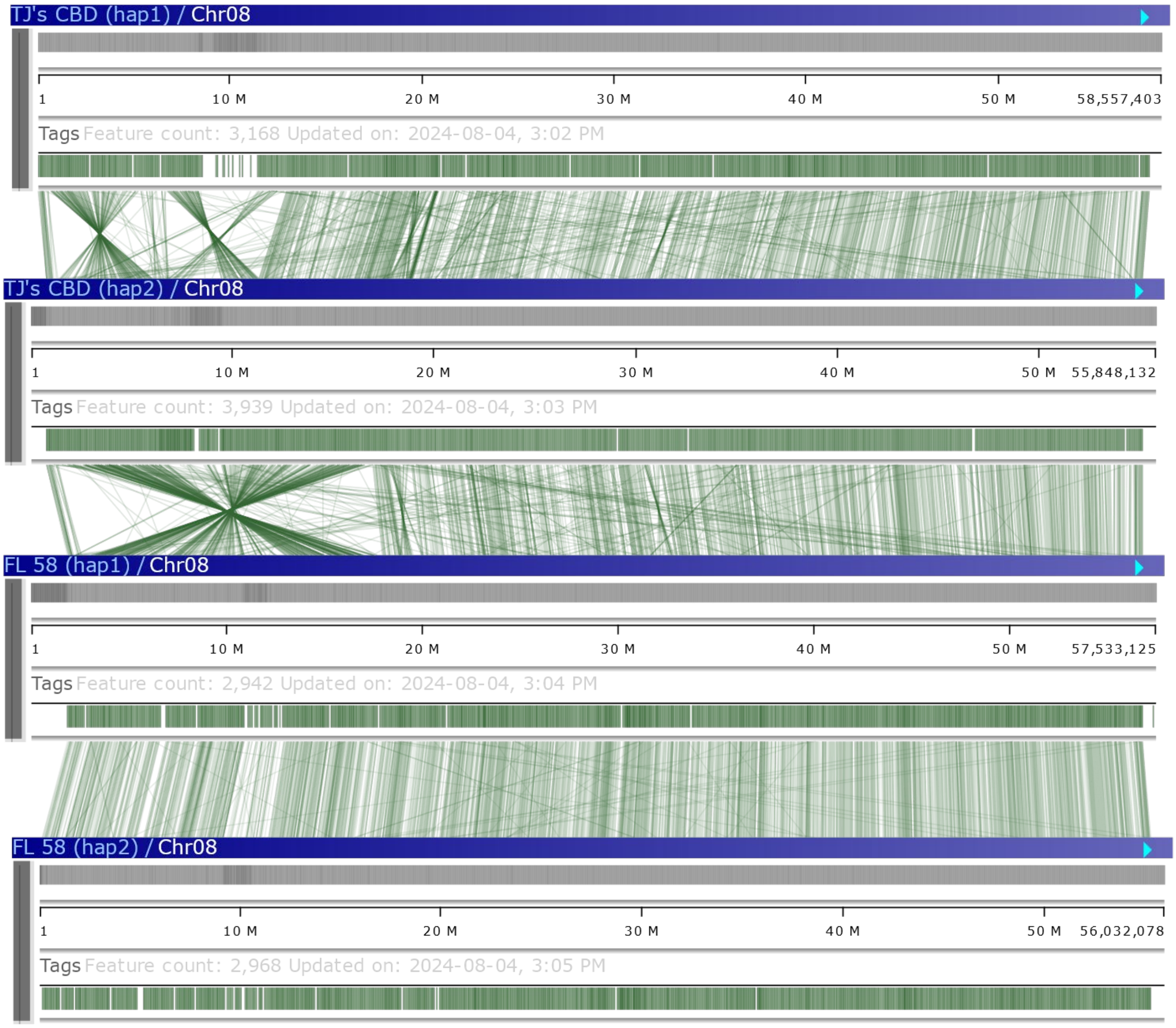

Supplementary Figure S2. Alignment of Chr08 from haplotypes of 'TJ's CBD' and 'FL 58' showing large structural variants in the first 20 Mb.

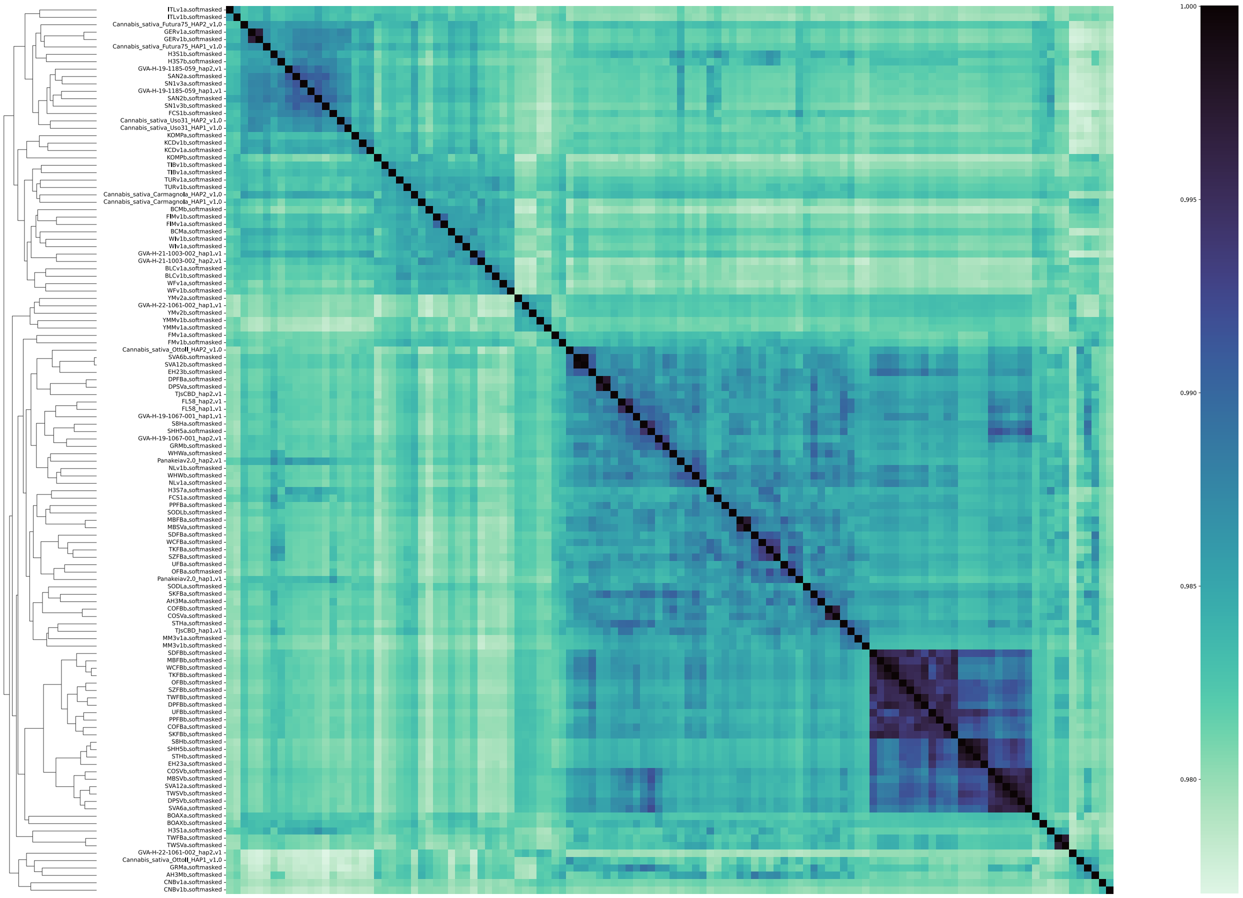

Supplementary Figure S3. Heatmap clustered by Jaccard similarity scores of 120 haplotypes from 60 phased *C. sativa* assemblies based on a whole-genome 31-mer PanKmer index.
